# Parathyroid Crisis: A Case of Elective Parathyroidectomy

**DOI:** 10.7759/cureus.40251

**Published:** 2023-06-11

**Authors:** Uchechi Adeniran, Beisi Ji, Israa Hussein, Lina Soni

**Affiliations:** 1 Internal Medicine, Woodhull Medical Center, Brooklyn, USA; 2 Internal Medicine, Downstate Health Science University of New York - Downstate Medical Center, Brooklyn, USA; 3 Endocrinology, Diabetes and Metabolism, Downstate Health Science University of New York - Downstate Medical Center, Brooklyn, USA; 4 Endocrinology, Downstate Health Science University of New York - Downstate Medical Center, Brooklyn, USA

**Keywords:** primary hyperparathyroidism, hypercalcemia, elective parathyroidectomy, parathyroid carcinoma, parathyroid crisis

## Abstract

A parathyroid crisis is characterized by a severe elevation in calcium, usually above 14-15 mg/dl alongside acute signs and symptoms of hypercalcemia. It is a rare but potentially life-threatening complication of primary hyperparathyroidism (PHPT). Among all primary hyperparathyroidism cases, parathyroid carcinoma accounts for only less than 1%. Although the definitive management is surgical parathyroidectomy, the exact timing of surgery is not well-established. We describe a case of a patient with abrupt onset of severe hypercalcemia who was managed medically and discharged for elective parathyroidectomy. This was because her workup was suspicious for parathyroid carcinoma, and there was a need to pursue a positron emission tomography (PET)-computed tomography (CT) scan to evaluate for other malignancies before proceeding with parathyroidectomy. The patient experienced the resolution of her symptoms of acute encephalopathy and improvement in her calcium levels from 22.3 mg/dl (8.8-10.2 mg/dl) on admission to 9.1 mg/dl on day 13 of hospitalization and discharge. In this report, we review the literature on the timing of parathyroidectomy in patients with a parathyroid crisis and how this has evolved over time with the use of new hypocalcemic agents. We discuss that parathyroidectomy performed emergently within 72 hours vs after 72 hours has not shown any significant impact on long-term survival in patients with parathyroid crisis. Moreover, medical management is crucial while waiting for surgery.

## Introduction

A parathyroid crisis is a rare endocrine emergency for which modern management has substantially improved outcomes in mortality and morbidity. A parathyroid crisis is defined as hyperparathyroidism with a marked elevation of parathyroid hormone (PTH), severe hypercalcemia (usually >14 - 15 mg/dl), and acute symptoms such as acute encephalopathy, anorexia, vomiting, cardiac arrhythmia, and acute renal injury [1-4]. More than 80% of PHPT cases are caused by a single parathyroid adenoma while parathyroid hyperplasia accounts for 10%, double adenoma for 4%, and parathyroid carcinoma for less than 1% [5].

The treatment of a parathyroid crisis is to normalize calcium levels. The initial and most important step in the management of hypercalcemia is to administer intravenous isotonic saline for extracellular volume expansion and promote hypercalciuria, with or without the usage of loop diuretics to prevent volume overload. Biphosphonate and calcitonin can also be used to treat parathyroid crises [5]. For parathyroid carcinoma, the definitive treatment remains surgical parathyroidectomy, however, the exact timing is not well-established.

Here, we present a case of a patient who presented with confusion and weakness, found to have a parathyroid crisis, who was medically optimized and discharged with follow-up for adequate surgical planning prior to elective parathyroidectomy.

## Case presentation

A 72-year-old female was brought in by Emergency Medical Services after she was found with confusion and extreme weakness in her apartment. The patient was last seen well by her family two days prior. She had a history of hypertension and hyperlipidemia and was taking antihypertensives medications (lisinopril and amlodipine) and atorvastatin. She also had a remote history of kidney stones. A review of systems was significant for polyuria, polydipsia, urinary incontinence, and muscle weakness.

Laboratory studies were significant for serum calcium level of 22.3 mg/dl (8.8-10.2 mg/dl, the baseline was 13.6 mg/dl one week prior), ionized calcium of 2.94 mmol/L (1.16-1.32 mmol/L), albumin of 4.6 g/dl (2.8-5.7 g/dl), chloride/phosphate ratio of 35. PTH of 1,077 pg/ml (15.0-65.0 pg/ml), 25-OH vitamin D of 56.68 ng/ml (>=30.00 ng/ml), 1, 25-OH vitamin D of 75.2 pg/ml (19.9-79.3 pg/ml), phosphorus of 2.9 mg/dl (2.5-4.5 mg/dl), estimated glomerular filtration rate (eGFR) of 37.0 ml/min/1.73m^2^ (>60 ml/min/1.73 m^2^, was normal one week prior), blood urea nitrogen (BUN) of 19.0 mg/dl (8.0-23.0 mg/dl), creatinine of 1.50 mg/dl (0.50-0.90 mg/dl, the baseline was 0.57 mg/dl one week prior). The patient was treated with intravenous normal saline, zoledronic acid, and calcitonin. We closely monitored her calcium levels and mental status. On day 4 of hospitalization, her calcium level went down to 10.1 mg/dl, and she was alert and oriented to person, place, and time and was back to her baseline function.

**Figure 1:**
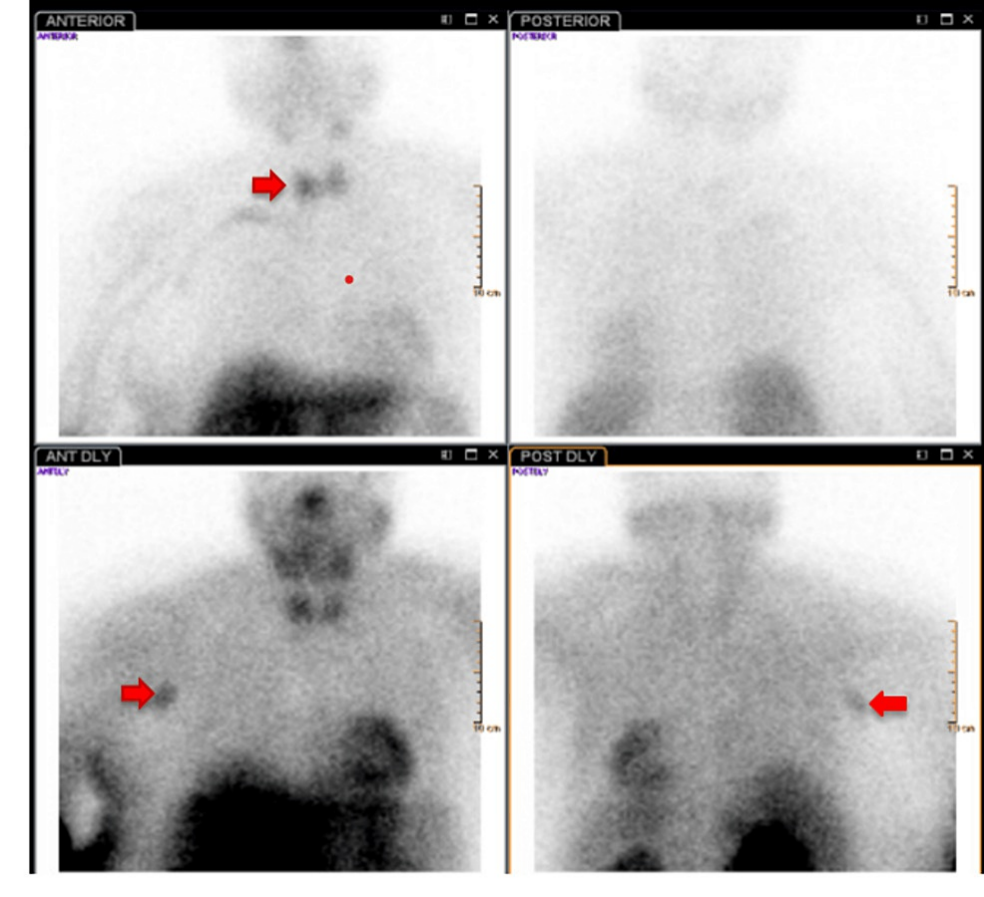
Nuclear medicine parathyroid scan Nuclear medicine parathyroid scan showing foci of abnormal tracer uptake in the region of the right and left poles suggestive of parathyroid hyperplasia and multiple foci adenomas and increased uptake in the right chest and right axilla suspicious of malignancy (arrow)

Ultrasound of the thyroid gland showed a heterogeneous multinodular thyroid gland with the largest nodule measuring 1.3 cm in the left thyroid lobe and 1.2 cm in the right thyroid lobe. The nuclear medicine parathyroid scan revealed foci of abnormal tracer uptake in the region of the right and left poles, suggestive of parathyroid hyperplasia and multiple foci adenomas and demonstrated increased uptake in the right chest and right axilla suspicious of malignancy (Figure 1). The computed tomography (CT) scan of the soft tissue neck with contrast showed bilateral retro thyroid nodules with enhancement - a 12 x 9 x 17 mm nodule in the left and a 15 x 11 x 20 mm nodule in the right, consistent with parathyroid adenomas.

The patient was discharged on day 13 with a calcium level of 9.1 mg/dl (Table 1), and she was discharged on cinacalcet 30 mg twice a day. She was instructed to follow up for a positron emission tomography (PET)-computed tomography (CT) scan of the whole body to assess for carcinoma metastasis (due to the parathyroid scan showing increased uptake in the right chest and right axilla suspicious of malignancy) and evaluate for other malignancy prior to surgical parathyroidectomy. The patient was also scheduled to follow up outpatient with Otolaryngology and Endocrinology.

**Table 1:** Routine biochemical profile, including the metabolic panel and blood count one week before admission, admission day 1, admission day 3, discharge, and latest follow-up NA: not available; WBC: white blood cell; BUN: blood urine nitrogen; GFR: estimated glomerular filtration rate; PTH: parathyroid hormone; PTHrP: parathyroid hormone-related protein

Variable	One week before admission	Admission day 1	Admission day 3	Discharge	Latest follow-up	Reference range
WBC (K/uL)	7.37	12.98	9.17	8.34	NA	4.5 – 10.9
Hemoglobin (g/dL)	12.6	14.5	12.9	12.0	NA	12.0 – 16.0
Calcium (mg/dL)	13.5	22.3	10.1	9.1	10.0	8.8 – 10.2
Albumin (g/dL)	4.0	4.6	4.1	4.0	4.3	2.8 – 5.7
BUN (mg/dL)	6.0	19.0	15.0	14.0	13.0	8.0 – 23.0
Creatinine (mg/dL)	0.64	1.5	1.19	0.95	0.67	0.5 – 0.9
GFR (ml/min/1.73m2)	>60.0	37.0	48.9	>60.0	>60.0	>60.0
Magnesium (mg/dL)	NA	1.48	1.72	1.65	NA	1.6 – 2.6
Phosphorus (mg/dL)	NA	2.9	2.1	3.1	NA	2.5 – 4.5
PTH (pg/mL)	NA	1077.0	NA	NA	61.0	15.0 - 65.0
25-Hydroxy Vitamin D (ng/mL)	NA	56.68	NA	NA	59.67	>= 30
1,25-Hydroxy Vitamin D (pg/mL)	NA	75.2	NA	NA	NA	19.9 – 79.3
PTHrP (pmol/L)	NA	<2.0	NA	NA	NA	<2.0

## Discussion

A parathyroid crisis is a potentially fatal complication of primary hyperparathyroidism. Severe hypercalcemia occurs in 1-2% of patients with primary hyperparathyroidism. Other complications, such as renal stones, renal insufficiency, proximal muscle weakness, brown tumor, and neuropsychiatric syndrome, are common in patients with parathyroid crises [6].

Although the parathyroid hormone was demonstrated in 1926 when Collip injected his dog with large doses of parathyroid extract, leading to a rise in serum calcium to 20 mg/100 ml and death, preceded by anorexia and vomiting, the parathyroid crisis was not recognized in man until 1932. A boy was treated with repeated injections of parathyroid extract for purpura, which resulted in persistent vomiting and lethargy, and the boy was close to dying. However, he recovered when the parathyroid extracts were discontinued [1]. The first case of the parathyroid crisis was reported by Hanes in 1938 and was caused by a parathyroid adenoma.

The management strategies for parathyroid crises have evolved over time with the advent of new hypocalcemic drugs. Although the definitive treatment remains surgical parathyroidectomy, the exact timing is not well-established. Wang and Guyton conducted a study that included 14 patients with a parathyroid crisis at Massachusetts General Hospital between 1964 and 1978 and concluded that prompt surgical intervention was the ideal treatment for parathyroid crises, preferably within 72 hours of acute onset of symptoms. However, medical treatment at the time was limited to the use of loop diuretics (furosemide), prednisone, potassium phosphate/sodium acid phosphate, and calcitonin in most patients [1]. Phytayakorn and McHenry, later on, described the use of bisphosphonate as an effective bridge to parathyroidectomy in patients with parathyroid crises [7]. Lew et al. carried out a study on the long-term results of parathyroidectomy for hypercalcemic crises. They reviewed 1055 consecutive patients who underwent parathyroidectomy at their institution from January 1, 1969, to October 31, 2004. They identified 43 patients with a hypercalcemic crisis. All the patients were noted to have received medical optimization with Intravenous fluids, loop diuretics, and hypocalcemic drugs (including bisphosphonate), followed by surgical excision. The average time between admission and parathyroidectomy was five days (range of 1-29 days). They concluded that there was no significant difference in long-term survival in patients treated with emergency parathyroidectomy in less than 72 hrs vs. parathyroidectomy done 72 hrs or more. Early surgical intervention was proposed [8,9]. Recent case series are in favor of early surgery after medical optimization rather than emergency surgery. Priority should be on medical management while workup and diagnostic tests are being pursued, and surgery should be expedited based on the patient's suitability and comorbidity [8].

In some patients, like our case, in which there was a need for more imaging studies to evaluate for malignancy owing to suspicious findings of increased uptake in the right chest and right axilla on the parathyroid scan; this raises the challenge of balancing between having all study reports available for optimal surgical planning vs. pursuing an emergent surgical intervention. Our patient was medically optimized, with improvement in serum calcium from 22.3 mg/dl on admission to 9.1 mg/dl and improvement in mental status and kidney function. The patient was subsequently scheduled to follow up for elective surgical parathyroidectomy after adequate imaging studies had been performed.

## Conclusions

A parathyroid crisis carries a high potential for mortality and morbidity if not treated promptly. With the advent of more medical therapeutic options, studies have shown no significant difference in long-term survival in patients who received an emergency parathyroidectomy performed within 72 hours vs. parathyroidectomy done after 72 hours. The focus is on the medical optimization of the patient while they await surgery.
